# 43745 A pilot survey to assess the practices, attitudes and beliefs around endotracheal aspirate culture use in a pediatric intensive care unit

**DOI:** 10.1017/cts.2021.537

**Published:** 2021-03-30

**Authors:** Anna Sick-Samuels, Anping Xie, Elliot Melendez, Jim Fackler, Aaron Milstone

**Affiliations:** 1 Johns Hopkins School of Medicine; 2 Connecticut Children’s Hospital

## Abstract

ABSTRACT IMPACT: Optimizing the use of endotracheal aspirate cultures (EACs) has the potential to improve the care of complex mechanically ventilated children by improving testing practices and avoiding unnecessary antibiotic treatment for false-positive results. OBJECTIVES/GOALS: An electronic survey has previously been employed to characterize the practices and attitudes around blood cultures among critically ill children. The objective of this work was to develop and pilot a new survey as a tool to understand practices and attitudes that could inform quality improvement initiatives to optimize EAC practices. METHODS/STUDY POPULATION: Informed by prior experience of diagnostic stewardship of EAC in other settings and using a similar structure to the blood culture practice survey, we developed an electronic self-administered survey sent to respiratory therapists, advanced practice providers, and physicians at the Johns Hopkins All Children’s pediatric intensive care unit. RESULTS/ANTICIPATED RESULTS: A total of 27 of 87 clinicians (37%) responded to the survey (22 respiratory therapists, 9 attending physicians and 1 advanced practice provider). Responses indicated samples are typically collected by respiratory therapists via in-line (endotracheal) or open suctioning (tracheostomy). Most respondents did not feel EACs could lead to unintended negative consequences (71%), agreed practices vary between people (89%), and felt an algorithm would help align the clinical team (79%). Most respondents agreed some clinicians may be reluctant to change practice (82%) and may not change practice due to concern for missing diagnosis of ventilator-associated pneumonia or tracheitis (78%). Surveillance cultures were not used in this unit and there were no prior EAC diagnostic stewardship efforts. DISCUSSION/SIGNIFICANCE OF FINDINGS: This survey captured practices, perceptions and barriers to changes that will inform the implementation of quality improvement initiatives to optimize EAC use in this unit. Future studies can consider utilizing an electronic survey to describe practice variation, clinician believes and attitudes about EAC testing in ventilated patients.

